# Dynamic graphene filters for selective gas-water-oil separation

**DOI:** 10.1038/srep14321

**Published:** 2015-09-23

**Authors:** Jihye Bong, Taekyung Lim, Keumyoung Seo, Cho-Ah Kwon, Ju Hyun Park, Sang Kyu Kwak, Sanghyun Ju

**Affiliations:** 1 Department of Physics, Kyonggi University, Suwon, Gyeonggi-Do 443-760, Republic of Korea; 2 School of Energy and Chemical Engineering, Ulsan National Institute of Science and Technology, Ulsan 689-798, Republic of Korea

## Abstract

Selective filtration of gas, water, and liquid or gaseous oil is essential to prevent possible environmental pollution and machine/facility malfunction in oil-based industries. Novel materials and structures able to selectively and efficiently filter liquid and vapor in various types of solutions are therefore in continuous demand. Here, we investigate selective gas-water-oil filtration using three-dimensional graphene structures. The proposed approach is based on the adjustable wettability of three-dimensional graphene foams. Three such structures are developed in this study; the first allows gas, oil, and water to pass, the second blocks water only, and the third is exclusively permeable to gas. In addition, the ability of three-dimensional graphene structures with a self-assembled monolayer to selectively filter oil is demonstrated. This methodology has numerous potential practical applications as gas, water, and/or oil filtration is an essential component of many industries.

In view of the prevalence of oil-based industries across the globe, high-efficiency water-oil filtration is essential to avoid oil wastage and protect the environment. Water-oil separation is a key step in a number of processes, namely oil drilling in exhausted oil fields, tertiary oil extraction, oil spill cleanups, and oil-bearing industrial wastewater purification. In this context, many studies have recently focused on the separation or absorption of water-oil^1^,^2^,^3^,^4^, mainly using mesh-type or foam-type polymers of nanostructured filters. Specifically, an oil-water separation method has been suggested based on the assembly of sodium silicate and TiO_2_ nanoparticles into two-dimensional (2D) stainless steel meshes^1^. Elsewhere, structured cone arrays have been investigated to collect micron-sized oil droplets in an effective manner^2^, and the oil sorption properties of polypyrrole have been enhanced through the assembly of functionalized graphene oxide^3^. Two-dimensional stainless steel meshes coated with polyacrylamide hydrogels have also been studied as a means to separate oil and water^4^.

The volume capacity of existing 2D meshes is however limited owing to the weight of larger mesh substrates. Two-dimensional meshes are furthermore susceptible to damage to any part of the mesh, do not readily filter vapor oil, and separate oil less efficiently than three-dimensional (3D) meshes. Indeed, large volumes are readily filtered through thicker 3D meshes without increasing the size of the substrate. Moreover, in terms of reliability, surface damage does not compromise the filtering ability of 3D meshes because of the underlying connected structure. Finally, the larger reaction surface presented by 3D meshes traps significantly larger volumes of oil vapor. Thus, in order to overcome many drawbacks of 2D filters, 3D structures for water-oil separation were investigated^5^,^6^,^7^,^8^.

Graphene has superior mechanical, thermal, and electrical characteristics than inorganic materials and its antibiotic properties inhibit the formation of bacteria^9^. These properties explain its widespread use for the adsorption of heavy metal ions or volatile organic pollutants, in fuel cells, and for energy storage^10^,^11^,^12^,^13^. In particular, 3D structured graphene has recently proved valuable for various devices, including batteries, supercapacitors, and sensors. In particular, graphene/NiO and graphene/Co_3_O_4_ supercapacitor electrodes have been prepared by creating 3D graphene networks through chemical vapor deposition^14^,^15^, and 3D graphene foam has been utilized as a monolithic and macro-porous carbon electrode for electrochemical sensing^16^.

In this study, 3D graphene structures were employed to selectively filter gas, water, and oil. To that end, three forms of graphene foams were prepared by adjusting their hydrophobicity *via* surface treatments (*i.e.*, with pristine graphene surfaces, those treated with O_2_ plasma, or covered with a self-assembled monolayer).

## Results and Discussion

**Figure 1:**
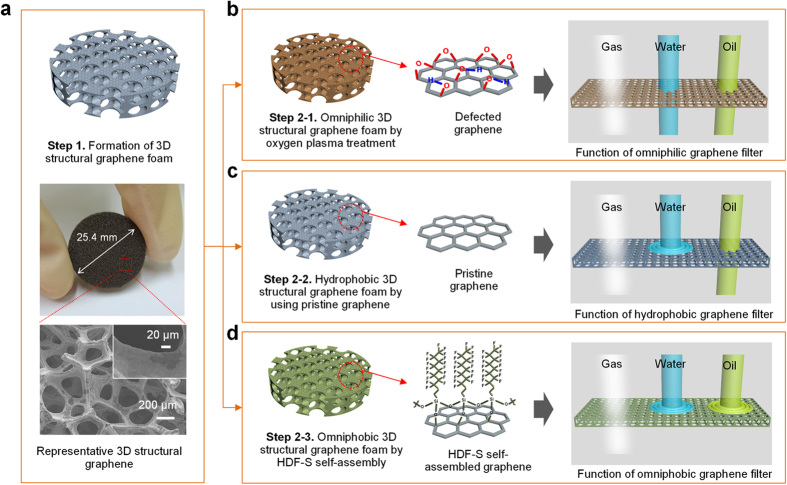
(**a**) Photograph of a 3D structural graphene foam 25.4 mm in diameter. FE-SEM image of the graphene foam with an average pore diameter of 150–200 μm. The inset shows a higher magnification image of the surface of a single graphene wire, in which the graphene laminated on the fracture surface is clearly observed. (**b–d**) Schematic illustration of three different types of graphene by using surface treatment and their selective filtering properties.

A schematic diagram and a photograph of a piece of 3D graphene foam 25.4 mm in diameter and 2 mm thick are shown in Fig. 1a, along with a surface field-emission scanning electron microscopy (FE-SEM) image of this foam, which reveals a 3D structure with pores of 150–200 μm diameter. The higher magnification image in the graphene-coated area (Fig. 1a inset) shows that the graphene has a perfectly laminated and rough surface (see Figure S5 in the Supporting Information for Raman spectra of 3D graphene foams). Schematic diagrams of the three different types of graphene foams used as selective filters are shown in Fig. 1b–d. These diagrams represent the omniphilic graphene foam obtained by surface O_2_-plasma treatment, the hydrophobic pristine graphene foam, and the omniphobic foam with self-assembled (heptadecafluoro-1,1,2,2-tetrahydrodecyl)trichlorosilane (HDF-S). As synthesized, 3D structural graphene is hydrophobic but the O_2_-plasma treatment generates defects on the graphene surface, rendering it omniphilic. For the omniphobic structure, the hydroxyl groups (−OH) on the graphene surface and the active group (trichlorosilane–SiCl_3_) of HDF-S form covalent bonds from which a strong siloxane network is built up. These three different types of graphene foam allow selective filtering of gas and/or liquids.

**Figure 2:**
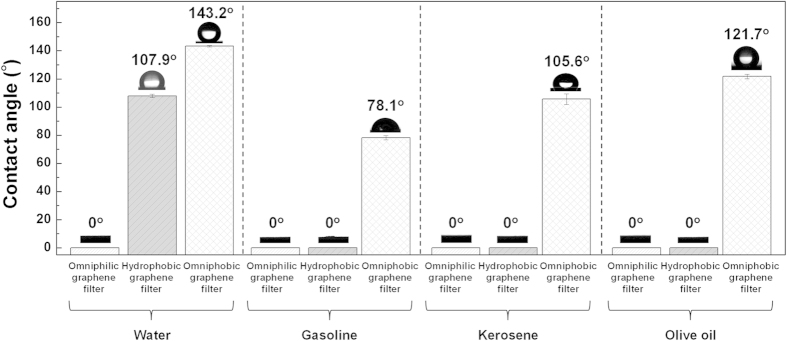
Wetting properties of omniphilic (O_2_-plasma-treated graphene foam), hydrophobic (pristine graphene foam), and omniphobic filters (graphene foam covered with self-assembled HDF-S) to different liquids.

Figure 2 shows the wetting properties of the three 3D-graphene structures (O_2_-plasma-treated, pristine, and with self-assembled HDF-S) to water, gasoline, kerosene, and olive oil. The average and standard deviation of five measurements performed for each sample are shown. The contact angle of all the liquids on the O_2_-plasma-treated graphene foam is 0°, which indicates omniphilic behavior. The contact angles of water on pristine graphene is 107.9 ± 1.1°, which contrasts with the 0° measured for gasoline, kerosene, and olive oil. The (hydrophobic) pristine graphene therefore blocks water only. On the other hand, the 3D graphene covered with self-assembled HDF-S is not only superhydrophobic (143.2 ± 0.5° contact angle with water, ~35° higher than measured for pristine graphene), but also oleophobic, with gasoline, kerosene, and olive oil contact angles of 78.1 ± 1.4°, 105.6 ± 3.8°, and 121.7 ± 1.7°, respectively. It is thereby constitutes an omniphobic filter.

**Figure 3:**
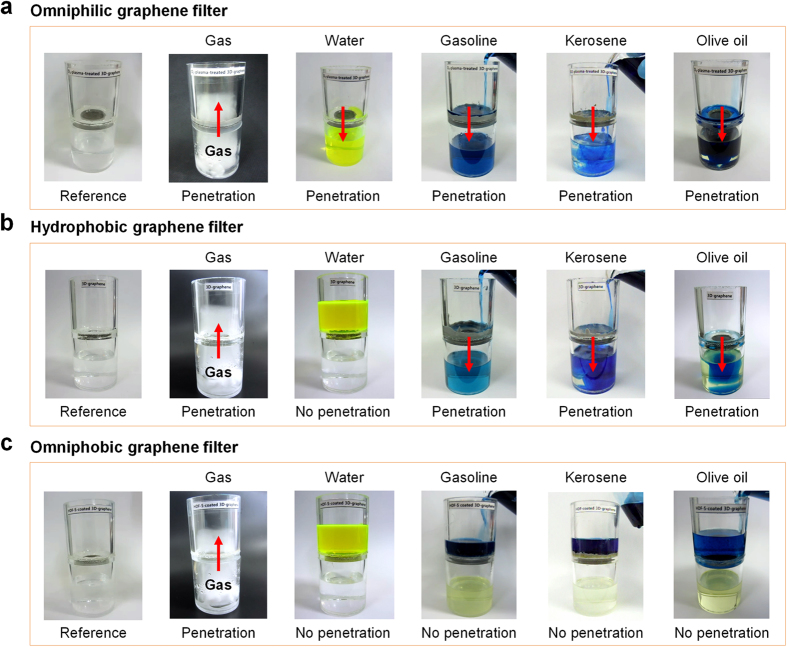
Gas/water/oil separation achieved with the omniphilic, hydrophobic, and omniphobic graphene filters coated on ~150–200 μm meshes. The graphene foams were fixed between a polypropylene (PP) tube and a PP beaker, and pure gas, water, or oil (the latter two with dye) were placed in the upper PP tube. Water permeates through the coated mesh, while the oil is repelled and retained in the upper tube.

Figure 3 demonstrates the transmission capabilities to gas, water, gasoline, kerosene, and olive oil of the omniphilic, hydrophobic, and omniphobic graphene filters. Each 3D filter was fixed between two PP beakers. Gas, water, and oil, mixed with dye to facilitate observation, were then injected through the upper beaker. Note that no external force was applied during the filtering process (see details in the Experimental Section and Supporting Information video). Gas, water, and oil rapidly permeate through the omniphilic graphene foam and drop into the beaker below (Fig. 3a). The hydrophobic graphene filter allows gasoline, kerosene, and olive oil to pass through but blocks water (Fig. 3b). The omniphobic graphene foam blocks all liquids (water and oils), with gas only permeating through the filter into the beaker below (Fig. 3c).

**Figure 4:**
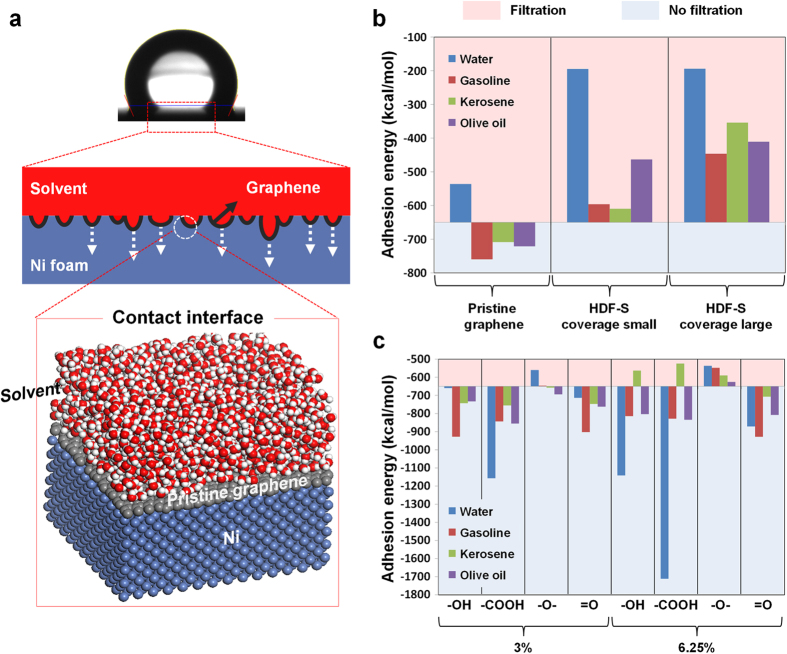
(**a**) Schematic diagram of typical solvent–substrate interface with water, graphene, and Ni molecules during the molecular dynamics simulation. (**b**) Adhesion energies—represented by bars overturned at −650 kcal/mol—of water, gasoline, kerosene, and olive oil to Ni substrates covered by pristine or omniphobic graphene. Solvents whose adhesion energy falls in the red region are filtered out, while those whose adhesion energy falls in the blue region pass through the filter. (**c**) Adhesion energies of water, gasoline, kerosene, and olive oil to Ni substrates covered with (3% and 6.25%) hydroxyl-, carboxyl-, epoxide-, and carbonyl-functionalized graphene.

The adsorption strength between each solvent and the substrate was investigated from a molecular perspective to elucidate different filtration behavior. First of all, the theoretical balance between surface tension and the gravitational force was calculated in a model pore system to estimate the maximum height of solvent that can be maintained by surface tension alone on a single pore (see Figure S1 in the Supplementary Information for a depiction of the model). The heights obtained are ~0.204 mm for water, ~0.206 mm for gasoline and kerosene, and ~0.207 mm for olive oil, much less than those used in the abovementioned tests (Fig. 2), suggesting that all these liquids should have passed through the filters. This highlights the selective filtering effects of the interactions between the solvents and the modified surfaces of each filter. The collective absorption through the interconnected pores in Fig. 4a is governed mainly by nonbonding interactions between the substrate surface and the solvent. The interaction energy was therefore estimated by performing molecular dynamics simulations (at 298 K) of a flat surface covered with solvent molecules (Fig. 4a). The adhesion energy calculated for each solvent to the substrate through different graphene filters is presented in Fig. 4b,c and Table S2, as calculated from van der Waals intermolecular interactions. The energy is negative in all cases, indicating favorable surface–solvent interactions. Since the adhesion (and gravitational) force pull the solvent molecules toward the substrate, and since the filters are highly porous, more negative interaction energies indicate greater solvent penetration. Here, we define an energy threshold for the penetration to occur in our systems by treating penetration-influencing factors such as 3D foam structure, its pore size and shape, and intrinsic properties of solvents as collective effect. Thus, by comparing the adhesion energy corresponding to the filtration phenomena in experiment, we determined the threshold energy, approximately −650 kcal/mol (*i.e.* ~0.22 N/m) for our systems. Based on this criterion, Fig. 4b clearly shows that pristine graphene blocks water but not gasoline, kerosene, or olive oil, while the graphene covered with HDF-S filters out all the solvents. Note that the relative degrees of filtration and penetration can also be estimated by the heights of bars. Since the role of the hydrophobic surface inducing solvents to cohere is crucial in this context, Fig. 4c highlights the effects of different functional groups. By applying the same threshold energy, a graphene surface covered at 3% or 6.25% with =O, −OH, or −COOH is permeable to all solvents other than kerosene, which is filtered out at 6.25% OH or −COOH. The fact that only kerosene is blocked suggests that the physical length and shape of the solvent molecules influence their filtration by the different graphene surfaces. Interestingly, Fig. 4c also shows that epoxide-functionalized graphene filters out all solvents at 6.25% coverage but is selective at 3% coverage, blocking only water. This indicates that the relative coverage of less attractive epoxide and more attractive *sp*^2^ carbon is the important factor governing the filtration of gasoline, kerosene, and olive oil.

## Conclusions

In summary, 3D graphene structures were fabricated whose wettability was controlled using a self-assembled monolayer. The hydrophobicity of the 3D graphene structures is substantially increased by the self-assembled HDF-S surface layer, with the water contact angle increasing from ~107.9° to ~143.2°. Raman analysis shows that the graphene in these structures is uniformly formed and X-ray photoelectron spectroscopy (XPS) measurements demonstrate that HDF-S self-assembles as desired on the graphene. The static apparent contact angle was measured on the graphene foams for liquids with different values of surface tensions, namely water, gasoline, kerosene, and olive oil. Water is selectively filtered by pristine (hydrophobic) graphene, while the HDF-S functionalized (omniphobic) graphene filter blocks all solvents. The porous structure of both filters (with 150–200 μm diameter pores) means, however, that they are both permeable to gas. Molecular dynamics simulations show that this selective filtration stems from the effects of different functional groups grafted onto the graphene. Indeed, while a high-percentage coverage of −OH and −COOH blocks kerosene, water is not filtered out with low-percentage epoxide coverage. This suggests a topic for further investigations, that the surface functionalization of graphene can be tailored to solve particular separation problems involving oil and water mixtures. This approach has numerous potential industrial applications. This study also provides a basis for future advances in gas-oil-water separation methods.

## Methods

### Preparation of 3D structural graphene foam

Ni foam with a 3D porous structure (pore size: 150–200 μm (120 pores per 25.4 mm), diameter: 25.4 mm, thickness: 2 mm, purity: 99.9%) was used as a catalyst and supporting frame for the fabrication of 3D structural graphene foams. The Ni foam was soaked in acetone and ulrasonicated for 3 min for cleaning and to remove organic contaminants from its surface. The clean foam was then annealed at 600 °C under flowing Ar (100 sccm) for 1 h. To grow graphene, the annealed Ni foam was placed in a chemical vapor deposition (CVD) chamber under H_2_ gas (100 sccm); the substrate temperature was then raised from room temperature to 1020 °C and kept at this temperature, first for 90 min under H_2_ gas and then for further 30 min under CH_4_ gas (20 sccm). The CH_4_ gas flow was then interrupted and the CVD chamber was cooled at a rate of 150 °C/min in H_2_ environment.

### Controlling the wettability of 3D structural graphene foams and preparing selective filters

Omniphilic graphene was obtained by O_2_-plasma treatment (30 W, 70 sccm, and 120 s). Graphene is hydrophobic as grown. In order to render it omniphobic, HDF-S (Gelest) was used to produce a trichlorosilane monolayer that self-assembles. The fabricated 3D graphene foam was immersed in a mixed solution of anhydrous toluene (99.8%) and silane precursor (3.0 mM), and trichlorosilane was left to self-assemble on the graphene surface in N_2_ ambient for 1 h. The graphene substrate covered with self-assembled HDF-S was then rinsed three times in anhydrous toluene and twice in isopropyl alcohol, before being left to dry for 1 h under ambient conditions.

### Optical and contact-angle measurements

The surface topology of the foams was observed by FE-SEM (S-4800, HITACHI) and the graphene was characterized by Raman spectroscopy. The presence of HDF-S and the surface chemical state of the graphene were verified by X-ray photoelectron spectroscopy (XPS, (PHI Quantera-II, Ulvac-PHI)). Contact angles (Phoenix 300, SEO) were measured by placing droplets (5 μL) of water, gasoline, kerosene, and olive oil on the surface of the samples under ambient conditions. For the gas penetration tests, the graphene foams were placed above dry ice in a closed bottle. To reveal the penetration of the different solvents, a water-soluble dye (fluorescin disodium salt, Samchun Pure Chemicals Co.) or an oil-soluble dye (Oil Blue N, dye content 96%, Sigma-Aldrich) was mixed into appropriate samples.

### Molecular dynamics simulations

Simulations were performed with four different solvents, namely water, gasoline (consisting of heptane, octane, and hexyl benzene), kerosene (consisting of n-hexadecane and n-eicosane), and olive oil (consisting of palmitic acid, stearic acid, oleic acid, and linoleic acid) (see Table S3 in the Supporting Information for the precise composition of each solvent and the structure of the molecules therein). The Ni substrate was covered with graphene, which was either simulated pristine or functionalized with HDF-S, -OH (hydroxyl), -COOH (carboxyl), -O- (epoxide), or =O (carbonyl) groups, with different coverages thereof (see Figure S2 in the Supporting Information for a detailed depiction of the modeled surfaces). The simulated surface was 42.60 Å × 49.19 Å across. Infinite layer systems were constructed by using periodic boundary conditions in the x- and y-directions, as shown in Figure S3. The simulations were performed in the *NVT* (*i.e.* canonical) ensemble at room temperature with 1 fs time steps. The systems were left to relax for 200 ps ahead of the 1 ns production runs used to calculate the adhesion energies. The values reported are averages from two independent simulations and the COMPASS force field was used throughout^17^,^18^,^19^.

## Additional Information

**How to cite this article**: Bong, J. *et al.* Dynamic graphene filters for selective gas-water-oil separation. *Sci. Rep.* **5**, 14321; doi: 10.1038/srep14321 (2015).

## Supplementary Material

Supplementary Information
