# Supplementary material for: Dynamic graphene filters for selective gas-water-oil separation
Source: Sci Rep. 2015 Sep 23;5:14321. doi: 10.1038/srep14321 (PMC4585788; doi:10.1038/srep14321)
Supplement: Supplementary Information [file srep14321-s1.doc]

**Supplementary Information**

**Dynamic graphene filters for selective gas-water-oil separation**

Jihye Bong^1^, Taekyung Lim^1^, Keumyoung Seo^1^, Cho-Ah Kwon^2^, Ju Hyun Park^2^, Sang Kyu Kwak^2,*^, and Sanghyun Ju^1,*^

^1^Department of Physics, Kyonggi University, Suwon, Gyeonggi-Do 443-760, Republic of Korea

^2^School of Energy and Chemical Engineering, Ulsan National Institute of Science and Technology, Ulsan 689-798, Republic of Korea

*Address correspondence to skkwak@unist.ac.kr, shju@kgu.ac.kr

**1. Force balance calculation**


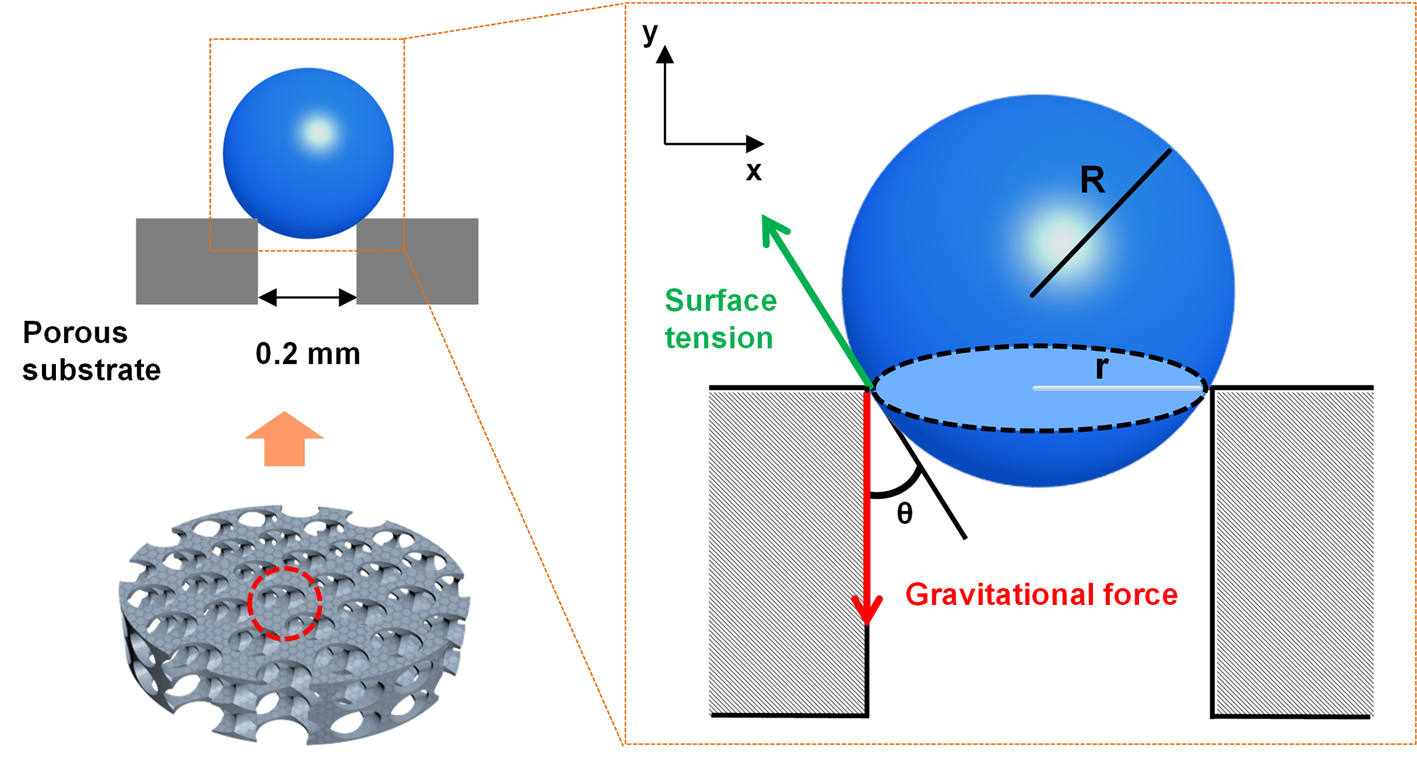


**Figure S1**. Abstract model of a porous substrate covered with solvent, upon which the force balance calculations are based.

An abstract pore model was used to estimate the maximum height of the solvent sustainable only by its surface tension. An average pore size of ~0.2 mm was chosen based on experimental observations. For the sake of calculation convenience, spherical solvent particles were considered. Under these conditions and at room temperature, the balance between the gravitational and the surface tension force for each solvent is derived as follows,

$$\rho g2\pi\left(\frac{r}{\cos\theta}\right)^{3}\left(\frac{2}{3}-\sin\theta+\frac{1}{3}\sin^{3}\theta+\cos^{2}\theta\sin\theta\right)=2\pi r\gamma\cos\theta$$

where *ρ* is the density of the solvent, *g* is the gravitational constant (*i.e.* 9.8 m/s^2^), *r* is the pore radius, *θ* is the angle between the pore wall and the line tangential to the surface of the solvent, and *γ* is the measured surface tension of the solvent. The gravitational and surface tension force appear respectively on the left and right term of Eq.. Solving Eq. for *θ* yields *R* via $r=R\cos\theta$. The values obtained in this way are listed in Table S1. These calculations show that even without considering the adhesive force acting in the –*y* direction, given the heights of liquid used experimentally, all the solvents should pass through the pores. However, in the experiment, the solvent was sustained by the filter. Therefore, we concluded that the adhesion force between the solvent and the inner surface of the pore was crucial for the filtering. We did not model the actual pore shape, as the pores were networked and large enough to prevent any confining effect from the pore surface on the solvent. Instead, we modeled the layered system of the solvent and the pore surface, including the Ni substrate (see Figure 4), which represented the side wall of the pore and the passing solvent, to estimate the adhesion energy.

**Table S1.** Mathematical parameters used in Eq.(1) for the model system depicted in Figure S1.

| **Name** | ***r* (m)** | ***ρ* (kg/m^3^)* *** | ***γ* (N/m)** | ***θ* (^o^)** | ***R* (m)** |
| --- | --- | --- | --- | --- | --- |
| **Water** | 1 × 10^−4^ | 1000 | 65.10 × 10^−3^ | 12.22 | 1.023 × 10^−4^ |
| **Gasoline** | 1 × 10^−4^ | 701.2^4^ | 23.83 × 10^−3^ | 14.39 | 1.032 × 10^−4^ |
| **Kerosene** | 1 × 10^−4^ | 776.8^5^ | 26.49 × 10^−3^ | 14.40 | 1.032 × 10^−4^ |
| **Olive oil** | 1 × 10^−4^ | 909.05^6^ | 30.11 × 10^−3^ | 14.51 | 1.033 × 10^−4^ |

**2. Surfaces in simulation systems**


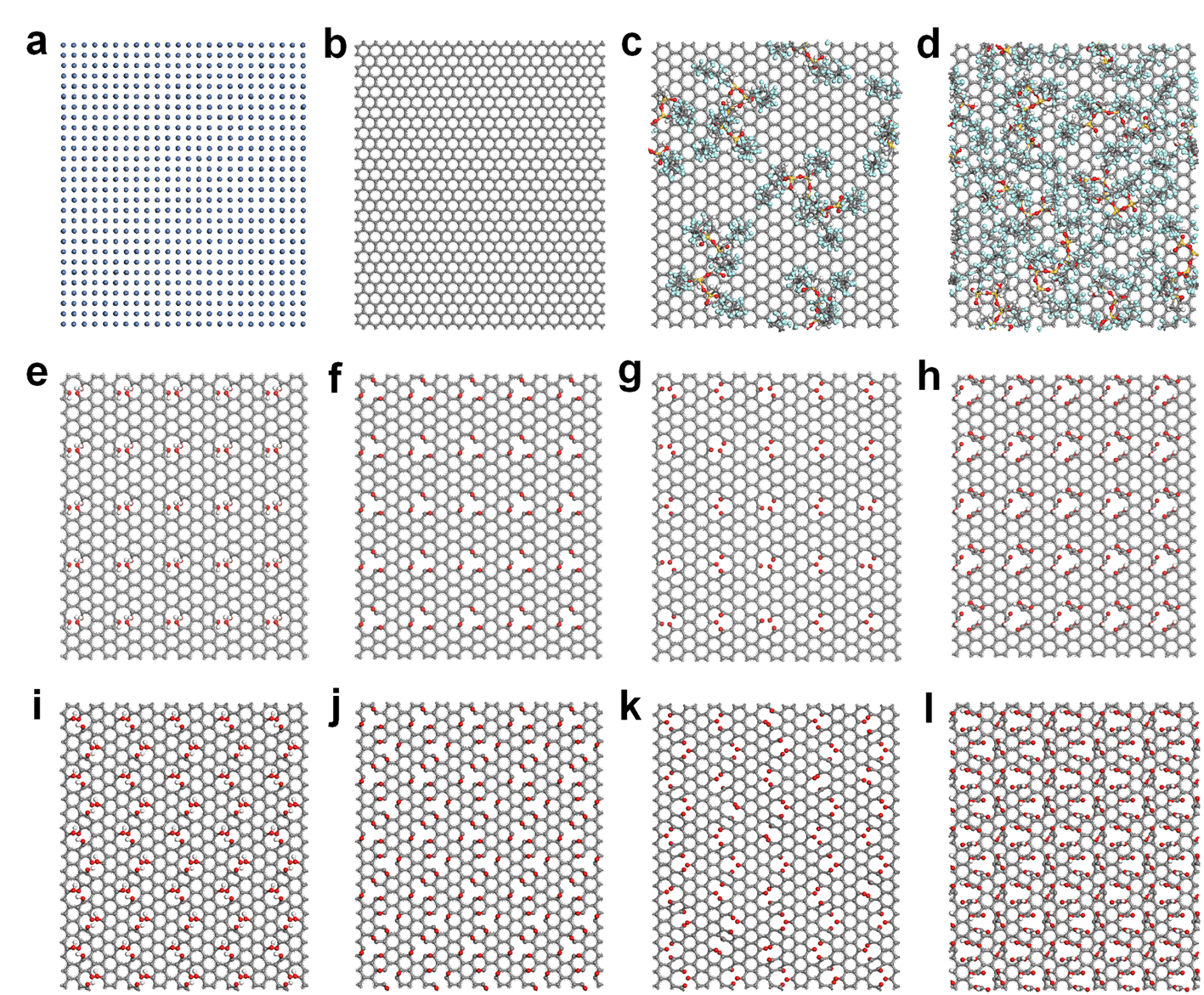


**Figure S2**. Top views of surfaces of (**a**) Ni. (**b**) pristine graphene. (**c**) graphene + low HDF-S coverage (25 molecules grafted). (**d**) graphene + high HDF-S coverage (45 molecules grafted). (**e**) graphene + OH (3%). (**f**) graphene + epoxide (3%). (**g**) graphene + carbonyl (3%). (**h**) graphene + carboxyl (3%). (**i**) graphene + OH (6.25%). (**j**) graphene + epoxide (6.25%). (**k**) graphene + carbonyl (6.25%). (**l**) graphene + carboxyl (6.25%). Each surface is 42.60 Å × 49.19 Å across. The percentages in parentheses are the proportions of *sp*^2^ carbon vacancy defects on the pristine graphene surface.

**3. Simulation systems**


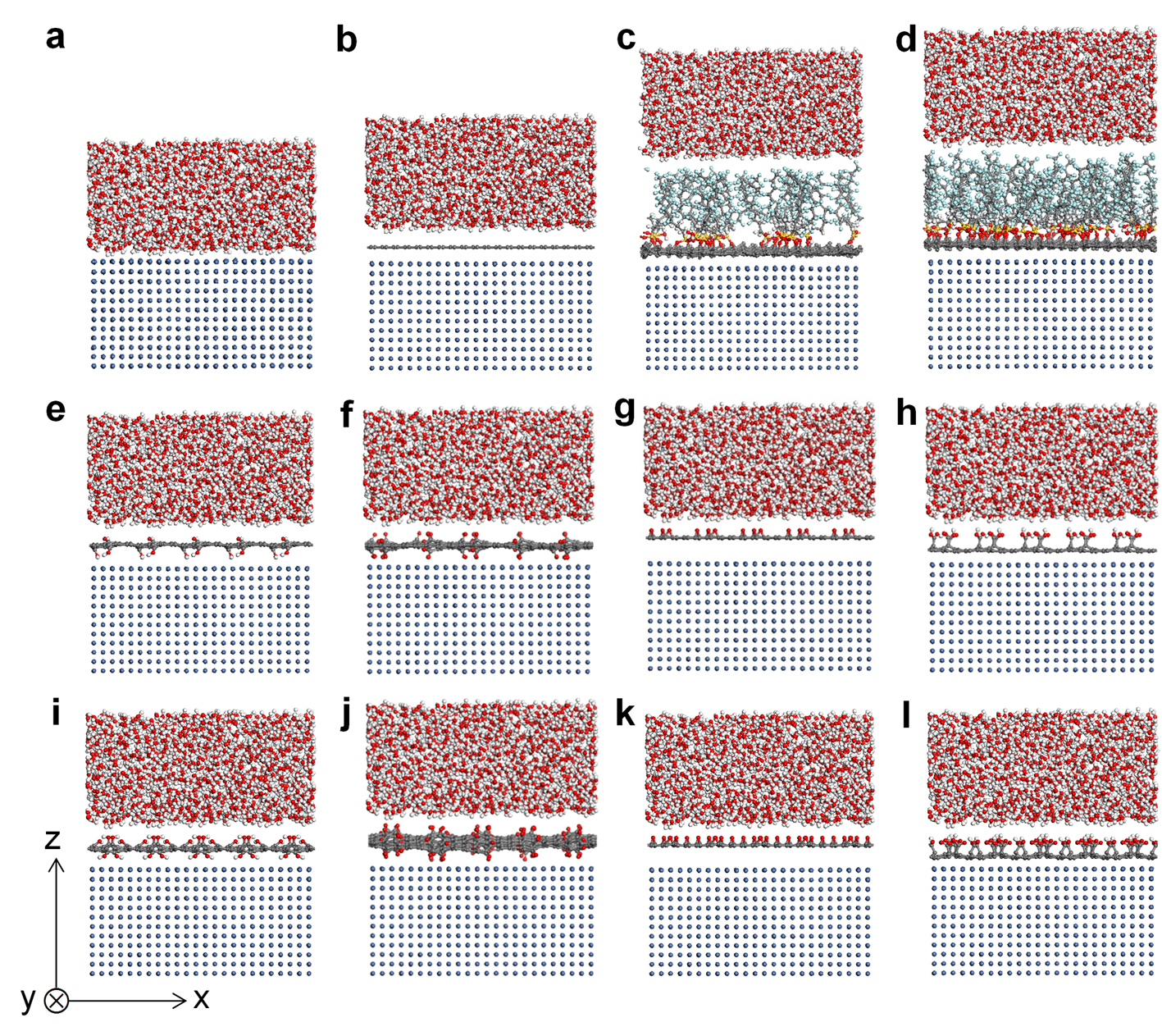


**Figure S3**. Side views of simulation systems with water. The substrate in each case is Ni. (**a**) direct contact between water and the substrate and (**b)**–(**l)** with intercalated layers of (**b**) pristine graphene; (**c**) graphene + low HDF-S coverage (25 molecules grafted); (**d**) graphene + high HDF-S coverage (45 molecules grafted); (**e**) graphene + OH (3%); (**f**) graphene + epoxide (3%); (**g**) graphene + carbonyl (3%); (**h**) graphene + carboxyl (3%); (**i**) graphene + OH (6.25%); (**j**) graphene + epoxide (6.25%); (**k**) graphene + carbonyl (6.25%); and (**l**) graphene + carboxyl (6.25%). The percentages in parentheses are the proportions of *sp*^2^ carbon vacancy defects on the pristine graphene surface. The vacuum slab (~200 Å thick) above the solvent is omitted for a clearer view of the simulation system. The simulations for the other solvents were conducted with the same systems, but with water replaced by gasoline, kerosene, or olive oil.

**4. Simulation results**

**Table S2**. Solvent–substrate adhesion energies (kcal/mol) with different graphene surfaces.

|  | **Water** | **Gasoline** | **Kerosene** | **Olive oil** |
| --- | --- | --- | --- | --- |
| **Ni** | −1100.57 | −2069.79 | −1949.58 | −1938.28 |
| **Graphene** | −536.41 | −759.78 | −708.87 | −721.07 |
| **HDF-S low coverage** | −194.87 | −596.21 | −609.56 | −463.29 |
| **HDF-S high coverage** | −193.98 | −446.41 | −354.02 | −410.62 |
| **-OH 3%** | −659.61 | −928.00 | −743.13 | −733.31 |
| **-COOH 3%** | −1156.86 | −843.65 | −755.39 | −855.36 |
| **-O- 3%** | −560.33 | −646.38 | −657.46 | −694.34 |
| **=O 3%** | −713.64 | −902.51 | −747.05 | −762.08 |
| **-OH 6.25%** | −1141.51 | −814.24 | −563.06 | −803.30 |
| **-COOH 6.25%** | −1710.95 | −828.34 | −524.48 | −834.23 |
| **-O- 6.25%** | −536.86 | −547.75 | −589.95 | −625.62 |
| **=O 6.25%** | −871.11 | −928.45 | −707.35 | −807.85 |

**5. Molecules in simulation systems**

**Table S3. Details of the molecules used in the simulation system. The compositions of gasoline, kerosene, and olive oil are from the literatures^1-3^. We chose the components shown below by considering the average number of carbon atoms from the carbon compound mixture of each solvent.**

| **Name** | **Components** | **Composition**  **(mol ratio)** | **Number of molecules** | **Chemical formula** | **Molecular structure** |
| --- | --- | --- | --- | --- | --- |
| **Water** | water | 1 | 1402 | H_2_O | 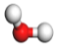 |
| **Gasoline^1^** | heptane | 0.3 | 48 | C_7_H_16_ | 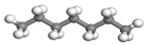 |
|  | octane | 0.64 | 101 | C_8_H_18_ | 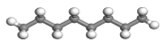 |
|  | hexyl benzene | 0.06 | 9 | C_12_H_18_ | 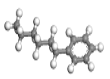 |
| **Kerosene^2^** | n-hexadecane | 0.2 | 66 | C_16_H_34_ | 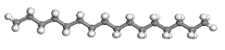 |
|  | n-eicosane | 0.8 | 17 | C_20_H_42_ | 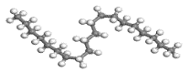 |
| **Olive oil^3^** | palmitic acid | 0.12 | 10 | C_16_H_32_O_2_ | 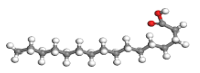 |
|  | stearic acid | 0.03 | 2 | C_18_H_36_O_2_ | 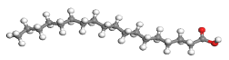 |
|  | oleic acid | 0.78 | 64 | C_18_H_34_O_2_ | 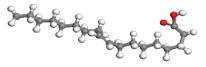 |
|  | linoleic acid | 0.07 | 6 | C_18_H_32_O_2_ | 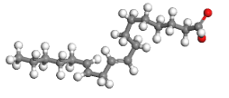 |

**6. The wettability of 2D films and 3D filters of graphene**


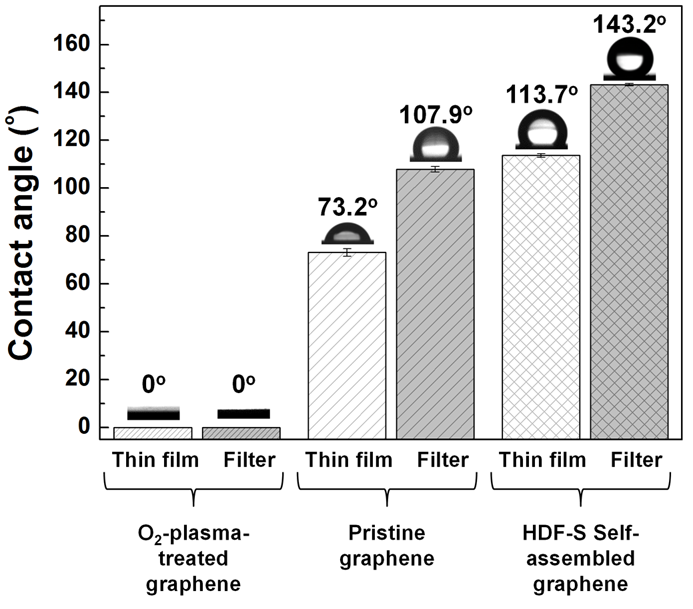


**Figure S4**. Wettability to water of graphene, either O_2_-plasma-treated (omniphilic), pristine (hydrophobic), or covered with self-assembled HDF-S (omniphobic), and as a 2D thin film or 3D filter.

Figure S4 shows the wetting properties of 2D films and 3D filters of graphene, O_2_-plasma-treated, pristine, or coated with self-assembled HDF-S. The O_2_-plasma-treated graphene is hydrophilic (*i.e.* the water contact angle thereon is 0°) in both thin-film and filter form. The contact angles of water on pristine graphene thin films and 3D filters are 73.2 ± 1.6° and 107.9 ± 1.1°, respectively. This indicates that the hydrophobicity of the filter substrate is higher, since the 3D Ni foam is rougher than the 2D film. For the graphene covered with self-assembled HDF-S, contact angles of 113.7 ± 0.7° and 143.2 ± 0.5° were measured for the 2D and 3D structures, respectively. This superhydrophobicity arises from the high roughness and the –CF_3_-terminated groups of the self-assembled monolayers.

**7. Raman spectroscopy**


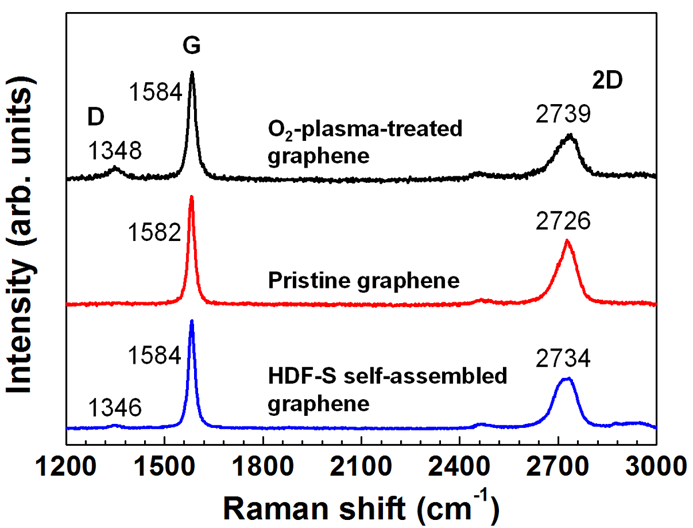


**Figure S5**. Raman spectra of 3D graphene foams, either O_2_-plasma-treated (omniphilic), pristine (hydrophobic), or covered with self-assembled HDF-S.

Figure S5 shows the Raman spectra obtained for the graphene foams, either O_2_-plasma-treated, pristine, or covered with self-assembled HDF-S. The spectrum for the pristine (hydrophobic) graphene foam, consists of a G peak at 1582 cm^–1^ and a strong 2D peak at 2726 cm^–1^. The G peak arises from doubly degenerate E_2g_ phonons at the center of the Brillouin zone through a first-order Raman scattering process. The D and 2D peak are respectively assigned to defects and its second-order peak, respectively^4,5^. The 2D/G peak intensity ratio (I_2D_/I_G_) is 0.60. The D peak is barely distinguishable in the spectrum for pristine graphene, highlighting the near absence of defects therein. On the other hand, the spectrum for the graphene covered with self-assembled HDF-S consist of a G, 2D, and D peaks at 1584 cm^–1^, 2734 cm^–1^, and 1346 cm^–1^, respectively; that is, the G and the 2D peak are respectively shifted by 2 and 8 cm^–1^ relative to their position in the spectrum for pristine graphene. In addition, the I_2D_/I_G_ ratio decreases (~0.46 vs. ~0.60). The shift of the G and the 2D peak, the decrease in the intensity of the latter, and the increase in intensity of the D peak all reflect the doping of graphene by the self-assembled monolayers^6^. For the O_2_-plasma-treated (omniphilic) graphene foam, G, 2D, and D peaks appears at 1584 cm^–1^, 2739 cm^–1^, and 1348 cm^–1^, respectively. The I_2D_/I_G_ ratio is ~0.43. This indicates that the O_2_ plasma treatment creates defects but does not severely damage the graphene. Here, “damaged graphene” refers to a torn sheet^7^, damaged such that the filtering capability induced by the O_2_ plasma treatment is lost. “Defect graphene” refers to graphene that has experienced amorphization of the *sp*^2^ lattice by the introduction of O as a substitutional impurity at carbon sites^8^, which causes the surface properties of graphene to change from hydrophobic to omniphobic.

**8. X-ray photoelectron spectroscopy**


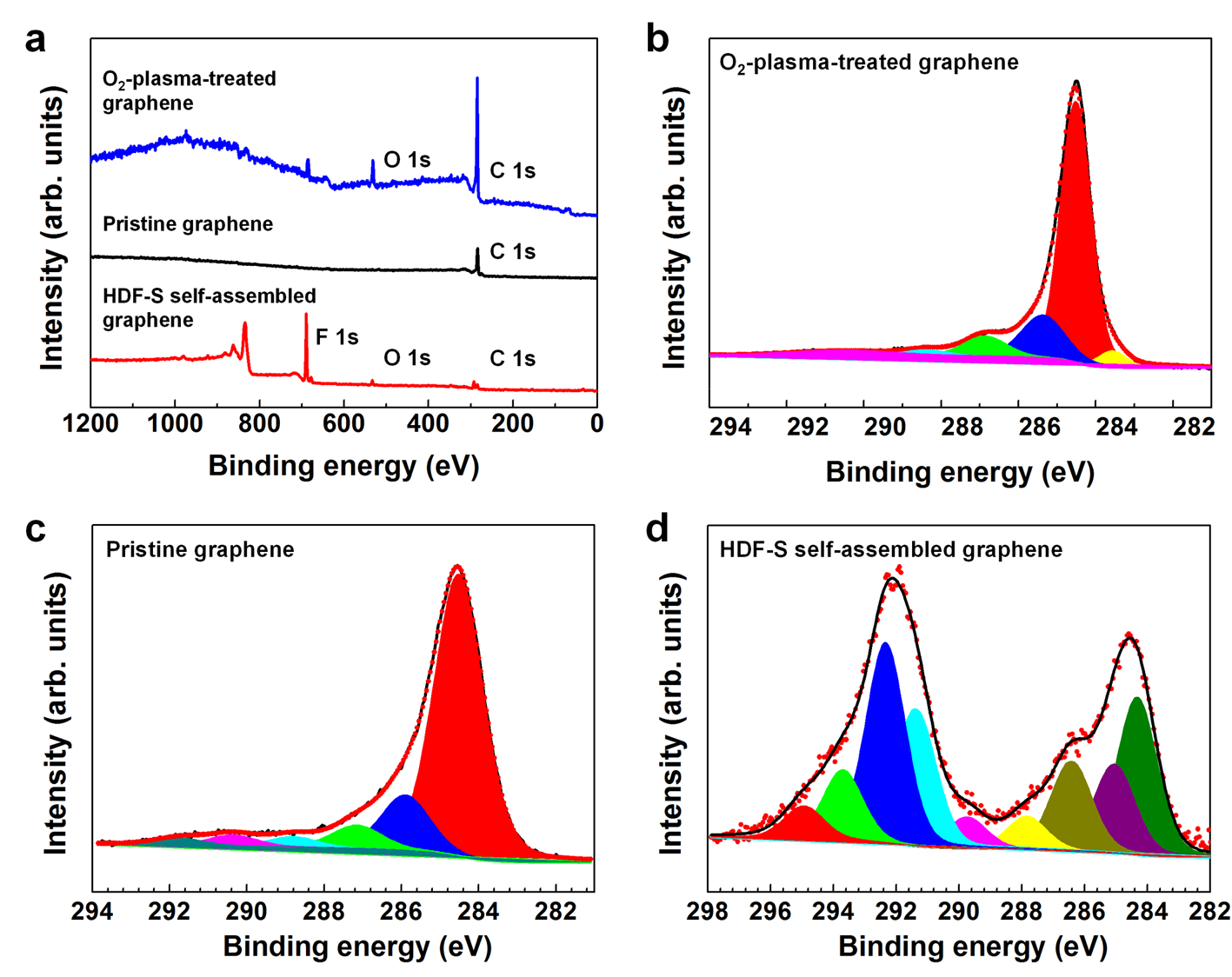


**Figure S6**. (**a**) XPS spectra of graphene foams, either O_2_-plasma-treated (omniphilic), pristine (hydrophobic), or covered with self-assembled HDF-S (omniphobic) in the full binding energy range (0–1200 eV). The F 1s peaks arise from fluorine composition. (**b**)–(**d**) C 1s core level XPS spectra of graphene (**b**) O_2_-plasma-treated, (**c**) pristine, and (**d**) covered with self-assembled HDF-S.

Figure S6a shows the XPS survey spectra recorded for graphene foams, either O_2_-plasma-treated, pristine, or covered with self-assembled HDF-S, with peaks corresponding to surface chemical groups with binding energies in the 0–1200 eV range. The main peaks for the O_2_-plasma-treated sample are the characteristic C 1s and an O 1s signals from graphene, at 284.0 eV and 532.3 eV, respectively. Deconvolution of the C 1s peak reveals a dominant C=C component (284.5 eV, 67.4% of integrated intensity), with smaller C–O (285.4 eV, 15.7%), C-O-C (286.9 eV, 7.4%), *sp*^2^ (283.6 eV, 3.4%), and OH–C–O (288.4, 2.0%) components (Fig. S6b). For pristine graphene, only the C 1s peak (284.0 eV) is observed. This is deconvoluted into C=C (284.5 eV, 72.6%), C–O (285.9 eV, 14.3%), C=O (287.2 eV, 5.8%), OH–C–O (288.8, 2.7%) peaks (Fig. S6c). For the foam covered with self-assembled HDF-S in contrast, a strong F 1s peak is observed at 689.0 eV along with the C 1s (284.9 eV) and O 1s (533.0 eV) peaks from graphene. Deconvoluting the C 1s peak reveals intense signals assigned to CF_2_ (291.4 eV, 292.3 eV, 40.3%) and CF_3_ groups (293.7 eV, 294.9 eV, 12.3%), with additional contributions from C=C (284.3 eV, 18.7%), C–C (285.1 eV, 10.5%), C-O-C (286.4 eV, 10.6%), C=O (287.8 eV, 4.0%), and O-C=O (289.7 eV, 3.8%) groups (Fig. S6d). These results indicate that HDF-S forms covalent bonds with the graphene surface, and thereby adheres strongly to the latter.

**9. Efficiency of selective separation**


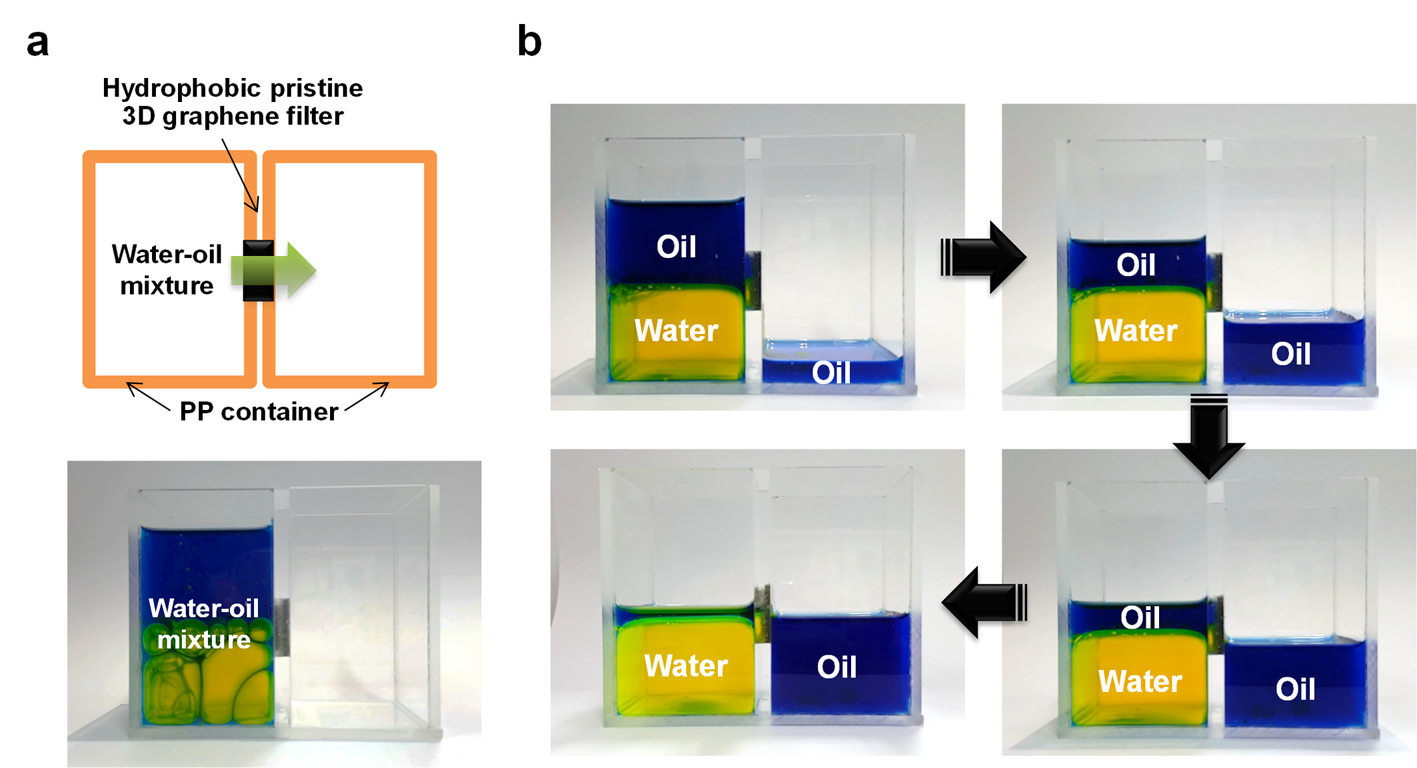


**Fig. S7.** (**a**) Schematic of the containers for oil-water separation. A hydrophobic pristine 3D graphene filter was installed on the side wall between two adjacent containers. (**b**) Photographs of the separation process for a mixture of water and olive oil (50 mL water mixed with yellow dye and 50 mL olive oil mixed with blue dye). The separation efficiency was 94.86%.

The efficiency of selective separation was investigated (Fig. S7). A mixture of water and oil (50 mL:50 mL) was poured into a container with a pristine hydrophobic 3D graphene filter positioned in the middle of the side wall of the container. As shown in the figure, only oil penetrated the pristine 3D graphene filter and moved to the adjacent container. The water-oil separation efficiency was calculated by the water rejection coefficient (R (%) = (1 – C_p_/C_0_) × 100)^9^. C_0_ and C_P_ are the water concentration in the original water-oil mixture and the collected oil after the separation, respectively. The hydrophobic-oleophilic pristine 3D graphene filter showed a separation efficiency of ~94.87%, comparable to the values found for previously reported filters (55–nearly 100%). Several of the reported filters showed high filtering efficiencies (>97%) because additional methods such as iteration^10^ and syringe pump^11^ were used to enhance the separation efficiency. We did not apply any additional methods to improve the filtering efficiency.

**References**

1. Wang, Z. F., Wang, L. S. & Fan, T. B. Densities and viscosities of ternary mixtures of heptane, octane, nonane, and hexyl benzene from 293.15K to 313.15K. *J. Chem. Eng. Data* **52,** 1886–1871 (2007).

2. Queimada, A. J., Quinones-Cisneros, S. E., Marrucho, I. M., Countinho, J. A. P. & Stenby, E. H. Viscosity and liquid density of asymmetric hydrocarbon mixtures. *Int. J. Thermophysics.* **24,** 1221–1239 (2003).
3. Gonzalez, C., Resa, J. M., Ruiz, A. & Gutierrez, J. I. Excess molar volumes of mixtures of hexane + natural oils from 298.15K to 313.15K. *J. Chem. Eng. Data* **42,** 339–341 (1997).

4. Casiraghi, C. *et al.* Raman spectroscopy of graphene `edges. *Nano Lett*. **9,** 1433–1441 (2009).

5. Ferrari, A. C. *et al.* Raman spectrum of graphene and graphene layers. *Phys. Rev. Lett*. **97,** 187401 (2006).

6. Park, J. *et al.* Work-function engineering of graphene electrodes by self-assembled monolayers for high-performance organic field-effect transistors. *J. Phys. Chem. Lett*. **2,** 841–845 (2011).

7. Song, M. S. & Cho, B. J. Investigation of the interaction between graphene and dielectrics. *Nanotechnology* **21,** 335706 (2010).

8. Nourbakhsh, A. *et al.* Bandgap opening in oxygen plasma-treated graphene. *Nanotechnology* **21,** 435203 (2010).

9. Cao, Y. Z. *et al.* Mussel-inspired chemistry and michael addition reaction for efficient oil/water separation. *ACS Appl. Mater. Interfaces* **5,** 4438–4442 (2013).

10. Lee, C. & Baik, S. Vertically-aligned carbon nano-tube membrane filters with superhydrophobicity and superoleophilicity. *Carbon* **48,** 2192–2197 (2010).

11. Lee, M. W. *et al*. Electrospun polystyrene nanofiber membrane with superhydrophobicity and superoleophilicity for selective separation of water and low viscous oil. *ACS Appl. Mater. Interfaces* **5,** 10597−10604 (2013).
